# The Relationship Between Anaphor Features and Antecedent Retrieval: Comparing Mandarin *Ziji* and *Ta-Ziji*

**DOI:** 10.3389/fpsyg.2015.01966

**Published:** 2016-01-05

**Authors:** Brian Dillon, Wing-Yee Chow, Ming Xiang

**Affiliations:** ^1^Department of Linguistics, University of Massachusetts AmherstAmherst, MA, USA; ^2^Department of Linguistics, University College LondonLondon, UK; ^3^Department of Linguistics, University of ChicagoChicago, IL, USA

**Keywords:** sentence processing, Mandarin Chinese, long-distance reflexives, working memory, referential processing

## Abstract

In the present study we report two self-paced reading experiments that investigate antecedent retrieval processes in sentence comprehension by contrasting the real-time processing behavior of two different reflexive anaphors in Mandarin Chinese. Previous work has suggested that comprehenders initially evaluate the fit between the morphologically simple long-distance reflexive “ziji” and the closest available subject position, only subsequently considering more structurally distant antecedents (Gao et al., 2005; Liu, 2009; Li and Zhou, 2010; Dillon et al., 2014; cf. Chen et al., 2012). In this paper, we investigate whether this *locality bias* effect obtains for other reflexive anaphors in Mandarin Chinese, or if it is associated specifically with the morphologically simple reflexive *ziji*. We do this by comparing the processing of *ziji* to the processing of the morphologically complex reflexive *ta-ziji* (lit. s/he-self). In Experiment 1, we investigate the processing of *ziji*, and replicate the finding of a strong locality bias effect for *ziji* in self-paced reading measures. In Experiment 2, we investigate the processing of the morphologically complex reflexive *ta-ziji* in the same structural configurations as Experiment 1. A comparison of our experiments reveals that *ta-ziji* shows a significantly weaker locality bias effect than *ziji* does. We propose that this results from the difference in the number of morphological and semantic features on the anaphor *ta-ziji* relative to *ziji*. Specifically, we propose that the additional retrieval cues associated with *ta-ziji* reduce interference from irrelevant representations in memory, allowing it to more reliably access an antecedent regardless its linear or structural distance. This reduced interference in turn leads to a diminished locality bias effect for the morphologically complex anaphor *ta-ziji*.

## Introduction

Anaphoric expressions such as pronouns (e.g., *him, he*), reflexives (e.g., *himself*), and anaphoric definite descriptions (e.g., *the boy*) have been widely studied in both linguistic and psycholinguistic traditions. Linguists have long been concerned with how the interpretation and syntactic distribution of referring expressions are determined (Chomsky, 1981; Heim, 1982; Elbourne, 2008; *a.o.*). Psycholinguists have studied anaphoric expressions both as a window into how comprehenders organize a text, and as a window into the working memory mechanisms that support sentence-level and discourse-level language comprehension (Kintsch, 1975; Gernsbacher, 1989; Greene et al., 1992; Myers and O'Brien, 1998; Foraker and McElree, 2011; Kush, 2013; Sturt, 2013; Dillon, 2014; *a.o*).

In the present work, we pursue a research question at the intersection of these two traditions. We ask how the form of a referring expression is related to the processing mechanisms that comprehenders use to assign it a referent. To this end, we contrast the processing of two closely related reflexive anaphors in Mandarin Chinese. Specifically, we compare the processing behavior of the morphologically simple reflexive *ziji* to that of the morphologically complex reflexive *ta-ziji*. In two self-paced reading experiments, we investigate the degree to which each anaphor exhibits a *locality bias*, a processing advantage (or preference) for syntactically local antecedents over more distant ones. Our empirical goal is to investigate the effect of morphological complexity on the processing of an anaphoric expression, with special attention to how morphology modulates the degree to which an anaphor will exhibit locality biases in processing. We interpret our results with respect to a theoretical model of anaphoric processing developed in other work (Dillon et al., 2014). To foreshadow our empirical and theoretical conclusions: our experimental findings suggest that morphologically complex anaphors in Mandarin Chinese show a diminished locality bias in comparison to morphologically simple ones, a finding that we attribute to how the processor makes use of the richer morphological feature content of morphologically complex anaphors in retrieving an antecedent from memory.

## Long distance reflexives and locality effects

In recent years, there have been a number of experimental investigations into the real-time processing behavior of the Mandarin Chinese long-distance reflexive *ziji*. *Ziji* is a morphologically simplex reflexive, literally meaning *self* (Huang et al., 2009). *Ziji* is a *long-distance* reflexive, unlike English reflexives which must be bound within their immediate tensed clause (their *binding domain*; Chomsky, 1981). Long-distance reflexives are so called because their binding domain is larger than their immediate tensed clause, although the exact size of their expanded domain varies across languages (see Büring, 2005). For Mandarin *ziji*, it appears that the binding domain is the entire root clause in which *ziji* is found (Tang, 1989; Xue et al., 1994; Huang and Liu, 2001; Büring, 2005; Huang et al., 2006, 2009). To take one example, in (1) *ziji* may be bound either by the subject of its immediate clause *Lisi*, or by the subject of the higher (root) clause *Zhangsan* (subscripts are used to indicate acceptable coindexation):

(1) 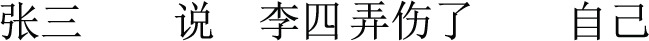  Zhangsan_j_ shuo Lisi_i_ nongshang-le ziji_i/j_
  Zhangsan say Lisi harm-perf self
  “Zhangsan says that Lisi harmed him/herself”

*Ziji* requires an animate antecedent (Tang, 1989; Xue et al., 1994; Huang and Liu, 2001), and receives an interpretation analogous to English reflexive forms. *Ziji* does not bear any overt morphological features, however, and so may take antecedents regardless of their gender, number, or person features.

Given the possibility of long-distance binding, it is interesting to note that many experimental studies have shown that comprehenders show a locality bias when processing *ziji*, preferring or more easily processing antecedents in their local clause over antecedents found in more distant clauses. For example, Li and Zhou (2010) conducted an ERP experiment in Mandarin, measuring the electrophysiological response to the anaphor “ziji” in examples like (2):

(2) a. 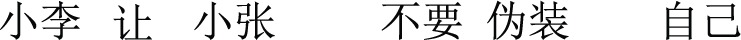  Xiaoli_i_ rang Xiaozhang_j_ buyao weizhuang ziji_?i/j_.
  Xiaoli ask Xiaozhang not disguise ziji.
  “Xiaoli asked Xiaozhang not to disguise himself.”
  b. 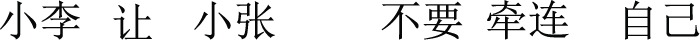  Xiaoli_i_ rang Xiaozhang_j_ buyao qianlian ziji_i/?j_.
  Xiaoli ask Xiaozhang not embroil ziji.
  “Xiaoli asked Xiaozhang not to embroil him.”

Li and Zhou observed a larger positivity (P300/P600) at *ziji* when the semantics of the verb created a bias toward a long-distance reading of the reflexive, as in (2b), compared to when the meaning of the verb biased comprehenders toward a local reading of the reflexive, as in (2a).

Cross-modal priming studies point to a similar advantage for local antecedents over long-distance antecedents. Gao and colleagues (Gao et al., 2005; Liu, 2009) presented participants with spoken sentences of the form in (1). Upon reaching the sentence-final *ziji*, participants were presented with a visual probe word. When the probe was presented immediately after the anaphor, participants recognized probes that were semantic associates of a local antecedent more quickly than they did probes associated with long-distance antecedents; this locality advantage disappeared or reversed at slightly longer SOAs (160 and 370 ms).

Using a different experimental paradigm, Chen et al. (2012) showed using self-paced reading that locally bound *ziji* was read more quickly than long-distance bound *ziji*. These authors leveraged the observation that *ziji* requires an animate antecedent to create the pair of experimental sentences in (3) (brackets are used to indicate tensed clause boundaries):

(3) a. 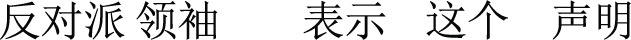  Fanduipai-lingxiu_i_ biaoshi [zhe-ge shengming_j_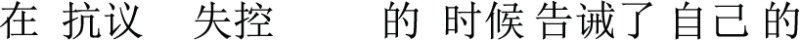  [zai kangyi_*k*_ shikong de- shihou] gaojie-le ziji_i/^*^j/^*^k_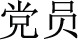  de dangyuan]
  opposition-leader say [this- cl announcement [at protest out.of.control time] warn- perf ziji de party.member]
  “The opposition leader said that this announcement warned his party members when the protest was out of control”
  b. 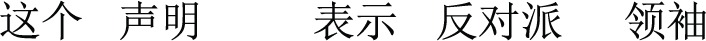  Zhe-ge shengming_i_ biaoshi [fanduipai- lingxiu_j_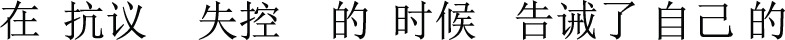  [zai kangyi_k_ shikong de-shihou] gaojie-le ziji_i/^*^j/^*^k_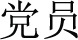  de dangyuan]
  this-cl announcement say [opposition-leader [at protest out.of.control time] warn-perf ziji de party.member]
  “The announcement said that the opposition leader warned his party members when the protest was out of control”

In these examples, *ziji* is the possessor of the direct object NP and it appears immediately after the embedded verb. In (3a), the only animate, c-commanding antecedent is *fanduipai-lingxiu*, “opposition leader.” Thus, in this example, *ziji* must take a long-distance antecedent in the immediately higher clause. In contrast, the embedded subject in (3b) is the only animate and c-commanding antecedent, and so *ziji* must take a syntactically local antecedent. In this paradigm, the difference in reading times between *ziji* in (3a) and (3b) is taken to indicate the difficulty of constructing a long-distance *ziji* interpretation in (3a). Chen and colleagues observed a small but reliable RT slow-down in reading times at the region following *ziji* (*de*) in (3a) relative to (3b), suggesting more difficulty in constructing a long-distance than local interpretation of *ziji*. This result was subsequently replicated in an eye-tracking while reading study, using direct object *ziji* in place of possessive *ziji*, and without an adverbial clause intervening between the subject and the verb (Jäger et al., 2015; Experiment 2).

Dillon et al. (2014) asked whether the locality bias associated with *ziji* reflected a difference in processing *speed* for accessing long-distance antecedents, or simply a difference in processing *accuracy* associated with long-distance antecedents. For example, it is possible that the memory trace of a distant antecedent is of relatively poor quality compared to that of a local antecedent, perhaps due to memory decay processes. Such a difference in the representational quality of the antecedent could have given rise to the locality bias effect observed in previous studies, without any difference in processing speed. Alternatively, it may be that comprehenders simply take more time to access a long-distance antecedent, such that local antecedent positions have a temporal advantage compared to more structurally or linearly distant positions. Simple response time measures cannot tease these possibilities apart (see McElree, 2006). In order to ask this question, Dillon and colleagues used the multiple-response speed-accuracy tradeoff (MR-SAT) technique to investigate sentences similar to those in (3). The (MR-)SAT technique involves eliciting behavioral responses at a series of pre-defined response deadlines. This allows the researchers to chart how accuracy on a response measure grows as a function of time, giving a full picture of the time-course of processing. Importantly, the resulting SAT function may be separated into independent measures of processing speed and processing accuracy. Dillon et al.'s results indicated that the difference between local and long-distance binding was reflected in the rate parameter of the SAT function, suggesting that comprehenders took longer to retrieve long-distance antecedents for *ziji* than local antecedents.

Thus, a growing body of evidence suggests that despite the formal possibility of long-distance antecedents for *ziji*, comprehenders experience relatively more processing difficulty when *ziji*'s antecedent is not local. Furthermore, this locality bias seems to reflect a temporal advantage for local antecedents over long-distance antecedents: comprehenders more rapidly access local antecedent positions than long-distance positions.

## *Ziji* and *ta-ziji*

Although previous research on *ziji* provides much evidence for a locality bias associated with *ziji*, it is not known how general this locality bias is. It is possible that a locality preference is a general property of long-distance reflexive anaphors. This might be expected if *ziji*'s locality bias was simply a reflection of the temporal or linear proximity of a local antecedent. In this case, we might expect all reflexive forms that can find an antecedent outside of their immediate clause to show comparable locality bias. On the other hand, it may be the case that the locality bias is rooted in some other specific property of *ziji*. For example, it may be the case that *ziji*'s lack of overt morphological features causes comprehenders to rely more heavily on positional cues when identifying an antecedent, which could lead to a preference for structurally local antecedents. If this is true, then we might expect the presence of locality bias effects to vary from anaphor to anaphor, depending on the surface form of the anaphor.

Mandarin grammar allows us to ask this question, because reflexive anaphors in Mandarin come in two forms: the morphologically simple, “bare” reflexive *ziji*, and morphologically complex anaphors. An example of a morphologically complex anaphor is *ta-ziji*, which consists of a third singular pronoun along with the bare reflexive (e.g. “he-self”). Other morphologically complex reflexives may be formed by combining other pronouns with *ziji* (e.g., *wo-ziji*, myself; *ni-ziji*, yourself), although here we focus on the third person singular form *ta-ziji*. *Ta-ziji* has a distribution that partially overlaps with *ziji*. For instance, when the antecedent of the anaphor is in the local clause, *ta-ziji* and *ziji* are interchangeable:

(4) 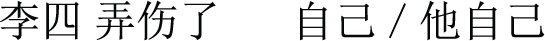  Lisi_i_ nongshang-le ziji_i_/ta-ziji_*i*_
  Lisi harm-perf self / 3sg-self
  “Lisi harmed himself”

The morphological differences between *ziji* and *ta-ziji* could lead to processing differences, because there are reasons to suspect that the addition of an overt pronoun to form a morphologically complex anaphor will yield richer cues for purposes of identifying an antecedent. First, the orthographic representation of the pronoun overtly provides gender and personhood cues: 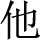 (t$\bar{\text{a}}$) is used for human male referents, 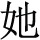 (t$\bar{\text{a}}$) is used for human female referents, and 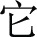 (t$\bar{\text{a}}$) is reserved for non-human or gender-neutral referents. These forms are distinguished orthographically, but they are not distinguished phonologically. In addition, the use of an overt pronoun is statistically more likely for gendered human antecedents than for non-human or gender neutral antecedents. A search of the Google Books corpus for simplified Chinese in the last 50 years reveals that approximately 73–80% of tokens of the third person singular pronoun refer to explicitly gendered human antecedents.

If this is correct, then we might say that *ta-ziji* has more features that can be used as cues to identify an antecedent when processing the reflexive. In particular, the addition of a pronominal form contributes humanness cues (i.e., [+human]) and gender cues. In contrast, the reflexive form *ziji* may only contribute animacy cues, because this is the only restriction that it places on potential antecedents. An interesting question to ask is whether the relatively more specified feature content of *ta-ziji* will lead to diminished locality bias for *ta-ziji* compared to *ziji*. If the locality bias associated with *ziji* reflects solely the influence of standard memory variables, such as decay or interference, then we might not expect the size or magnitude of the effect to vary with the surface form of the anaphor. If, on the other hand, the surface form of the anaphor contributes additional cues to identifying an antecedent, then locality bias might be diminished or eliminated for anaphors whose surface form bears more overt features. Thus, in the present study we aim to provide a head to head comparison of the locality bias associated with morphologically simple and morphologically complex anaphors, in an attempt to determine how generally locality bias is in the processing of anaphors in Mandarin.

Unfortunately, a direct comparison of the locality effects for *ziji* and *ta-ziji* is somewhat complicated by the fact that they do not have identical syntactic distributions. In contrast to *ziji*, the size of *ta-ziji*'s binding domain is a matter of some controversy. Huang et al. (2009) reported that it must be bound within its immediate tensed clause, like English *himself*. However, Pan (1998, 2000) argued that the binding domain of *ta-ziji* is fixed by the closest accessible animate antecedent, such that *ta-zij* can be bound outside of its local clause if the local subject is inanimate. What is clear is that *ta-ziji* places greater restrictions on long-distance antecedents than does *ziji*, and for this reason it is sometimes classified as a purely local reflexive in Mandarin (Huang et al., 2009). Because of the lack of clarity in the binding domains associated with these two reflexives, it is not ideal to compare these reflexives in the same embedding configurations used in previous studies (Chen et al., 2012; Dillon et al., 2014; Jäger et al., 2015).

Instead, we compared the behavior of *ziji* and *ta-ziji* in environments where they do have reliably overlapping distributions. For both *ziji* and *ta-ziji* the c-command relation that regulates binding in English (Chomsky, 1981) appears to be too restrictive. Instead, antecedents that do not strictly c-command these anaphors may be grammatically available, as in (5) (Tang, 1989):

(5) 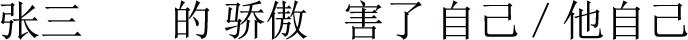  Zhangsan_i_ de jiao'ao_j_ hai-le ziji_i/^*^j_/ ta-ziji_i/^*^j_
  Zhangsan de arrogance harm-perf ziji / 3sg-ziji
  “Zhangsan's arrogance harmed him.”

In (5), the antecedent *Zhangsan* is embedded inside the subject, and hence does not c-command the anaphor. Nonetheless, in this configuration it is available to bind the reflexive. The structural relationship between Zhangsan and *(ta-)ziji* in (5) is referred to as *subcommand* (Tang, 1989; Huang and Tang, 1991). An NP is said to subcommand the anaphor if it is contained within an NP in subject position that c-commands or subcommands the anaphor (Tang, 1989).

However, it is important to note that subcommanding antecedents are not freely available. Instead, a subcommanding antecedent is only available when no animate c-commanding or subcommanding antecedent is structurally closer to *ziji*. Thus, when the subject head noun is animate, subcommanding antecedents are grammatically blocked as in (6):

(6) 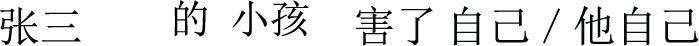  Zhangsan_i_ de xiaohai_j_ hai-le ziji_*i/*j*_/ ta-ziji_*i/*j*_
  Zhangsan de son harm-perf ziji / 3sg-ziji
  “Zhangsan's son harmed himself.”

As *ziji* and *ta-ziji* distribute similarly in subcommanding environments, we may compare the processing of *ziji* and *ta-ziji* in configurations like (7):

(7) a. 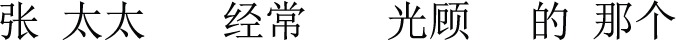  [Zhang taitai_i_ jingchang guanggu de] na-ge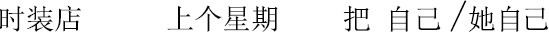  shizhuangdian_j_ shang-ge-xingqi ba ziji_i/^*^j_ / ta-ziji_i/^*^j_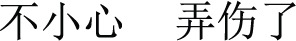  bu xiaoxin nongshang-le.
  Mrs. Zhang often visit de that-cl boutique last-week ba self / 3sg-self not careful harm-perf.
  “The boutique that Mrs. Zhang often visits carelessly harmed her last week.”
  b. 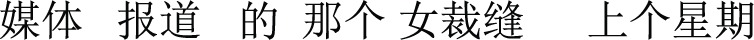  [Meiti_i_ baodao de] na-ge nü-caifeng_j_ shang-ge-xingqi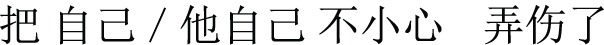  ba ziji_*i/*j*_ / ta-zij_*i/*j*_ bu xiaoxin nongshang-le.
  Media report-on de that-cl seamstress last-week ba self / 3sg-self not careful harm-perf.
  “The seamstress that the media reported on carelessly harmed herself last week.”

These examples have an object extracted relative clause (e.g., “that the media reported on”) modifying a subject noun (e.g., *na-ge nü-caifeng* “that seamstress”). This structure creates two subject positions that could in principle bind an anaphor: the *local* subject position inside the matrix clause, and a *distant* subject position inside the relative clause. Given the licensing constraints on *ziji* and *ta-ziji*, we expect that both the local subject *na-ge nü-caifeng* “that seamstress” in (7b) and the long-distance subject *Zhang taitai* “Mrs Zhang” in (7b) should be grammatically accessible antecedents. However, these two antecedents differ in their structural and linear distance from the reflexive. The subcommanding antecedent *Mrs. Zhang* in (7a) is a long-distance antecedent because it is linearly and structurally more distant from the anaphor than the local antecedent *na-ge nü-caifeng* in (7b). For this pair of conditions, then, a locality effect should present as increased reading times on the anaphor in (7a) compared to (7b).

We now present two experiments that investigate *ziji* and *ta-ziji* in Mandarin Chinese. Our goal in these experiments was to compare the processing profile of these two anaphors on a number of different dimensions. First, and most importantly, we ask whether both *ziji* and *ta-ziji* show locality effects of comparable magnitude in online sentence comprehension. In addition, we ask whether the processing of both *ziji* and *ta-ziji* is equally affected by the presence of multiple feature-matched antecedents. Previous research suggests that the presence of multiple, feature-matched antecedents may cause processing difficulty (the *multiple match effect* of Badecker and Straub, 2002), although this effect has not be observed in all studies (e.g., Clifton et al., 1999).

## Experiment 1

### Participants

Forty-one students from the University of Maryland community participated in the experiment. One participant was removed prior to analysis due to low comprehension question accuracy (see below). All participants were native Mandarin Chinese speakers from mainland China, and all had normal or corrected-to-normal vision. They were paid $10 for their participation in the experiment. Experimental sessions lasted approximately 45 min. Participants gave informed consent under an experimental protocol approved by the University of Maryland Institutional Review Board.

### Stimuli

We created stimuli with the sentence structure in (7). We orthogonally manipulated the animacy of the local subject position and the embedded subject position, yielding four experimental conditions. These conditions are summarized in (8).

a. *LOCAL MATCH:*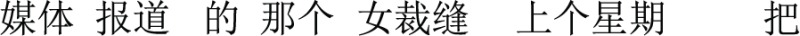  Meiti/ baodao de/ na-ge/ nücaifeng/ shang-ge-xingqi/ ba/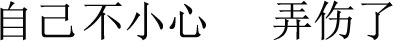  ziji/ bu xiaoxin/ nongshang-le.
  Media/ report-on de / that-cl / seamstress/ last-week/ ba/ self/ not careful/ harm-perf.
  “The seamtress that the media reported on carelessly harmed herself last week.”

b. *DISTANT MATCH:*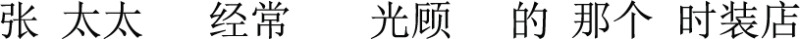  Zhang taitai/ jingchang guanggu de/ na-ge/ shizhuangdian/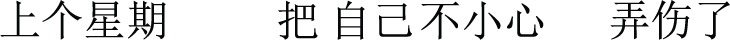  shang-ge-xingqi/ ba/ ziji/ bu xiaoxin/ nongshang-le.
  Mrs. Zhang/ often visit de / that-cl / boutique/ last-week/ ba/ self/ not careful/ harm-perf.
  “The boutique that Mrs. Zhang often visits carelessly harmed her last week.”

c. *MULTIPLE MATCH:*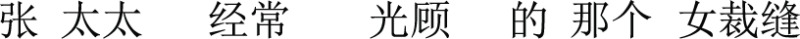  Zhang taitai/ jingchang guanggu de/   na-ge/ nücaifeng/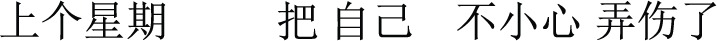  shang-ge-xingqi/ ba/ ziji/ bu xiaoxin/ nongshang-le.
  Mrs. Zhang/ often visit de / that- cl/ seamstress/ last-week/ ba/ self/ not careful/ harm-perf.
  “The seamstress that Mrs. Zhang often visits carelessly harmed her/herself last week.”

d. *NO MATCH:*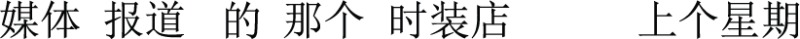  Meiti/ baodao de/ na-ge/ shizhuangdian/ shang-ge-xingqi/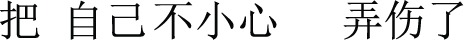  ba/ ziji/   bu xiaoxin/ nongshang-le.
  Media/ report-on de/ that-cl/ boutique/ last-week/ ba/ self/ not careful/ harm-perf.
  “The boutique that the media reported on carelessly harmed her last week.”

The paradigm employed here thus followed Chen et al. (2012), Dillon et al. (2014), and Jäger et al. (2015) in using animacy to manipulate the binding possibilities for *ziji*. In the *LOCAL MATCH* and *DISTANT MATCH* conditions, the antecedent of *ziji* is the animate subject. In the *NO MATCH* condition, there is no intra-sentential antecedent for *ziji*. In the *MULTIPLE MATCH* condition, the local subject *na-ge nü-caifeng* “that seamstress” is the only grammatically available antecedent of *ziji*. In this condition, the animate local subject blocks access to the distant subject Mrs. Zhang; therefore, the interpretation of *ziji* is not ambiguous in the *MULTIPLE MATCH* condition (see Tang, 1989).

The primary comparison of interest for the present purposes is the difference in reading times between the *LOCAL MATCH* and *DISTANT MATCH* conditions at the anaphor. The *MULTIPLE MATCH* and *NO MATCH* conditions were included for two reasons. First, the factorial manipulation of the animacy of the two subject positions allows us to dissociate effects of interest from simple effects of local or distant subject animacy. Second, the inclusion of the *MULTIPLE MATCH* conditions allows us to estimate any reading time effects associated with multiple feature-matched antecedents (the multiple match effect, Badecker and Straub, 2002). The inclusion of the *NO MATCH* condition serves as a control. This allows us to evaluate whether readers were indeed attempting to find an antecedent for *ziji*; if this is the case, then the failure to find an appropriate antecedent in this condition should lead to longer reading times.

The distant (sub-commanding) antecedent position was always the subject of an object relative clause that modified the main clause subject. Owing to the head-final order of noun phrases in Mandarin Chinese, this embedded subject (distant antecedent) is both structurally and linearly further away from the anaphor than the main clause subject (local antecedent). The local antecedent always followed the relative clause verb and the relativizing particle *de*. In order to construct plausible and natural sentences, the predicate inside the relative clause was different for animate (8a-c; e.g., “that Mrs. Zhang often visits”) and inanimate (8b-d; “that the media reported on”) relative clause subjects. The main clause predicate was constant across conditions.

In order to avoid having the critical word (the anaphor) in sentence-final position, the *ba* construction was used, because this construction has an S-ba-O-V word order (in contrast to the canonical SVO word order of Mandarin). A temporal adverbial was placed between the main clause subject (the local antecedent) and the *ba*-marked *ziji* to ensure that they were not adjacent to each other. A manner adverbial was placed between *ziji* and the main clause verb in order to provide an extra spillover region.

Eighteen sets of experimental items were produced, and distributed into four lists in a pseudo-Latin square fashion. They were combined with 77 fillers, including materials from an unrelated experiment, for a total of 95 sentences. The ratio of acceptable-to-unacceptable sentences varied slightly from list to list due to the pseudo-Latin square procedure, but remained between 83 and 85% acceptable. The fillers included 10 sentences that contained *ba* followed by non-anaphoric NPs in order to prevent participants from associating *ba* with *ziji* within the experiment.

### Procedure

Sentences were presented using a moving-window self-paced reading paradigm, using the Linger software (Rohde, 2003). Each sentence was presented in black characters on a white screen, and no sentence was more than one line long. All sentences were presented using simplified Chinese characters. The sentences were segmented into 9 regions according to native speaker intuitions about where best to insert boundaries [regions are indicted by slashes in (8)]. This procedure resulted in regions that ranged from one character (e.g., *ba*) to 6 characters (e.g., *yishuticaoguanjun*, “gymnastics champion”).

Sentences initially appeared as a series of dashes that obscured the entire sentence. Participants pressed the space bar to present the first region, and each subsequent space bar press masked the current region and triggered presentation of the subsequent region. Reaction times between button presses were recorded. After approximately 50% of the filler sentences, a Yes/No comprehension question was presented in its entirety on the screen, and participants were instructed to press one of two buttons to indicate their response. Feedback was given for incorrect responses. The critical *ziji* sentences never were followed by comprehension questions.

In the analyses below we refer to the region containing *ziji* as the *critical region*, and the region that follows (e.g., *bu xiaoxin*) as the *spillover region*.

### Statistical analysis

We performed a single statistical analysis over the pooled data in Experiments 1 and 2, which we present after Experiment 2. Reaction time data from both experiments were analyzed using linear mixed effects models with three critical experimental contrasts. Taking the *LOCAL MATCH* condition as the baseline, we defined the *Locality contrast* as the difference between the *DISTANT MATCH* condition and the *LOCAL MATCH* condition. As in previous studies (Chen et al., 2012; Dillon et al., 2014; Jäger et al., 2015), this contrast is interpreted as the penalty associated with long-distance binding of the anaphor. We further defined the *Multiple Match contrast* as the difference between the *MULTIPLE MATCH* condition and the *LOCAL MATCH* condition; this contrast is interpreted as the penalty associated with having multiple NPs that matched the features of the anaphor. Lastly, we defined the *No Antecedent contrast* as the difference between the *LOCAL MATCH* condition and the *NO MATCH* conditions. Each of these contrasts was coded with treatment coding, treating *LOCAL MATCH* as the baseline. These experimental contrasts were shared across Experiments 1 and 2. In addition to these fixed effects, we further included *Experiment* as a fixed effect with treatment coding, treating Experiment 1 as the baseline. Lastly, to test for differences in our experimental contrasts across experiments, we included terms for the interaction of *Experiment* with each experimental contrast.

Because our linear mixed effects models assume a normally-distributed response, we applied the Box-Cox procedure to reaction times at the regions we analyzed to determine a transformation that would yield a normally distributed response variable (Box and Cox, 1964). This procedure suggested a transformation in-between a negative reciprocal transform and a logarithmic transformation. Exploratory data analyses revealed that the qualitative pattern of results did not change under different transformations, and so we present the results of linear mixed effects models fit to logarithmically transformed reading time data. We adopted a “maximal” random effects structure, including random intercepts and random slopes for all fixed effect parameters within both subject and item grouping factors where possible (Barr et al., 2013). If the full model failed to converge, we removed random correlations but retained random slopes for all fixed effects.

Because of the pseudo-Latin square procedure, the number of sentences within each condition was not balanced within subjects. To test for any effects this imbalance may have had, we performed log-likelihood ratio tests of models with and without a fixed effect for experimental list. If log-likelihood tests indicated an effect of *list*, we performed further model comparisons to determine if the effect of *list* interacted with our experimental fixed effects.

In constructing the materials, we did not attempt to control the length or frequency of the subject noun phrases within items. However, as it has been shown that antecedent frequency is inversely correlated with reading times on anaphoric expressions (Van Gompel and Majid, 2004), we included antecedent frequency and antecedent length for both embedded and matrix subject positions as fixed effect control predictors in all mixed effects models. Antecedent frequency was estimated using the SUBTLEX Chinese corpus (Cai and Brysbaert, 2010). Many of our antecedent phrases were noun-noun compounds that were unattested in the corpus (e.g., *laladuiyuan*, “cheerleading squad member”). If the entire compound phrase was unattested, we used frequency of the head noun. Length was entered into the model as the number of characters of the head noun in the subject phrase. Both antecedent frequency and length were centered before being entered into the model.

Analysis was performed for three regions of the experimental sentences: the pre-critical region *ba*, the critical region *ziji*, and the spillover region [e.g., *bu xiaoxin* in (8)].

### Results

#### Offline judgments

Prior to Experiment 1, we gathered offline acceptability judgments of all experimental materials. All experimental stimuli, including fillers, were entered into the online experimental platform IbexFarm (Drummond, 2011). Twenty-two native Mandarin speakers were recruited from Beijing Normal University. They were directed to a web address that hosted the offline naturalness judgment questionnaire and they were asked to rate each experimental stimulus on a scale from 1 (not natural) to 7 (very natural).

The results of this offline judgment study are presented in Table 1. These data were analyzed using linear mixed effects modeling, with fixed effects for matrix subject animacy, distant subject animacy, and their interaction. This analysis revealed a main effect of local NP animacy (Est = −1.09 ± 0.25, *t* = −4.3), and an interaction of local and distant NP animacy (Est = −1.29 ± 0.38, *t* = −3.45). There were lower acceptability ratings for both conditions with a local inanimate subject (*DISTANT MATCH* and *NO MATCH*). However, a *post-hoc* comparison between these two conditions revealed that average ratings were significantly lower in the *NO MATCH* condition than in the *DISTANT MATCH* condition ($\bar{\text{x}}=-0.9$, 95%CI: [−1.4, −0.4]).

**Table 1:** **Mean acceptability ratings in Experiment 1**.

***LOCAL MATCH***	***DISTANT MATCH***	***MULTIPLE MATCH***	***NO MATCH***
5.2 (0.3)	4.4 (0.3)	4.8 (0.3)	3.5 (0.3)

Parentheses represent standard error by participants, corrected for between-participant variance (Bakeman and McArthur, 1996).

#### Comprehension

One participant was removed from further analysis due to low accuracy (less than 70% accurate). After this exclusion, accuracy on the comprehension questions in Experiment 1 averaged 87% across participants, indicating that the participant attended to the stimuli. Across participants, accuracy ranged from 73 to 98%.

#### Reading times

**Figure 1:**
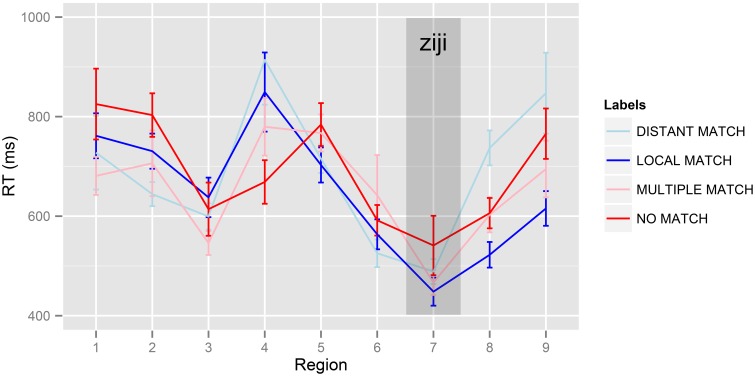
**Mean reading times per region in Experiment 1**. Error bars represent standard error by participants, corrected for between-participant variance (Bakeman and McArthur, 1996). 1:*Zhang taitai* 2:*jingchang guanggu de* 3:*na-ge* 4:*shizhuangdian* 5:*shang-ge-xingqi* 6:*ba* 7:*ta-ziji* 8:*bu xiaoxin* 9:*nongshang-le.*

Raw mean reading times in Experiment 1 are presented in Table 2 and in Figure 1.

**Table 2:** **Mean reading times per region in Experiment 1**.

	**1**	**2**	**3**	**4**	**5**	**6**	**7**	**8**	**9**
*LOCAL MATCH*	762 (45)	731 (35)	638 (40)	849 (80)	703 (35)	564 (30)	448 (28)	522 (26)	615 (35)
*DISTANT MATCH*	728 (74)	644 (24)	599 (27)	914 (90)	715 (29)	525 (28)	489 (25)	737 (35)	847 (81)
*MULTIPLE MATCH*	681 (38)	706 (66)	546 (24)	780 (58)	767 (37)	642 (81)	467 (25)	603 (35)	695 (57)
*NO MATCH*	825 (71)	803 (44)	614 (53)	669 (44)	784 (43)	592 (31)	541 (60)	606 (30)	766 (51)

Parentheses represent standard error by participants, corrected for between-participant variance (Bakeman and McArthur, 1996). Region labels are as follows: 1:Zhang taitai 2:jingchang guanggu de 3:na-ge 4:shizhuangdian 5:shang-ge-xingqi 6:ba 7:ziji 8:bu xiaoxin 9:nongshang-le.

### Discussion

The results of the offline judgment experiment revealed that raters assigned lower ratings to sentences where there was not a local antecedent for *ziji*. The lowest ratings were given to the *NO MATCH* condition, presumably reflecting the unacceptability that results from the lack of an intra-sentential antecedent. Interestingly, the *DISTANT MATCH* condition was rated lower than the *LOCAL MATCH* and *MULTIPLE MATCH* conditions. This penalty is consistent with the presence of a locality effect. This conclusion is supported by independent evidence that the acceptability of grammatical sentences is reliably modulated by the length of a binding dependency (Sprouse et al., 2011). However, it is also possible that this reflects relative unacceptability that results from having an inanimate matrix subject. Critically for the present purposes, the *DISTANT MATCH* condition was rated as more acceptable than the *NO MATCH* condition, consistent with the claim that the distant subject is grammatically accessible as an antecedent for *ziji* (Tang, 1989; Huang and Tang, 1991).

The results of the self-paced reading experiment suggest a locality effect in the reading times, with the *DISTANT MATCH* condition being read 41 ms more slowly than the *LOCAL MATCH* condition at the critical region, and 215 ms more slowly in the spillover region. If reliable, this finding would extend the locality bias effect observed in previous experiments to the subcommanding configuration tested here (Chen et al., 2012; Dillon et al., 2014; Jäger et al., 2015). The data also suggest numerically smaller effects of the *Multiple Match contrast* and the *No Antecedent contrast*: in the spillover region, reading times were 81 ms longer in the *MULTIPLE MATCH* condition than in the *LOCAL MATCH* condition, and 84 ms longer in the *NO MATCH* condition than in the *LOCAL MATCH* condition.

Before further interpreting the data in Experiment 1, we present the results of Experiment 2.

## Experiment 2

Experiment 2 was identical to Experiment 1 in all major respects, except that Experiment 2 investigates the processing of the complex anaphor *ta-ziji*.

### Participants

Seventy students from the University of Maryland community participated in the experiment. All participants were native Mandarin Chinese speakers from mainland China, and all had normal or corrected-to-normal vision. They were paid $10 per hour for their participation in the experiment. Participants gave informed consent under an experimental protocol approved by the University of Maryland Institutional Review Board.

### Stimuli

The materials were largely identical to those from Experiment 1. Two important changes were made to these materials. First, all instances of *ziji* were replaced with *ta-ziji*. The materials were additionally modified so that within an experimental item set, the animate nouns in each position were of the same gender. This was done to ensure that both NPs in the *MULTIPLE MATCH* condition matched the features of the reflexive. This change was necessary because *ta* orthographically marks gender. Half of the revised materials had male nouns, and the other half had female nouns.

All other aspects of the stimuli, including the fillers and comprehension questions, were identical to Experiment 1.

### Procedure

The experimental procedure was identical to Experiment 1.

#### Offline judgments

As in Experiment 1, we gathered offline naturalness judgments of all experimental materials prior to running Experiment 2. Collection of judgments and recruitment of participants proceeded in the same fashion as the offline pre-test for Experiment 1. Twenty-six native Mandarin speakers were recruited from Beijing Normal University.

The results of the offline judgment study are presented in Table 3. Linear mixed effects modeling revealed only an interaction of local and distant NP animacy (Est = −1.88 ± 0.38, *t* = −4.15). This interaction was driven by low ratings in the *NO MATCH* and *MULTIPLE MATCH* conditions. There was no appreciable difference between the ratings of the *LOCAL MATCH* and *DISTANT MATCH* conditions.

**Table 3:** **Mean acceptability ratings in Experiment 2**.

***LOCAL MATCH***	***DISTANT MATCH***	***MULTIPLE MATCH***	***NO MATCH***
**5.1 (0.3)**	**5 (0.3)**	**4.1 (0.3)**	**4 (0.3)**

Parentheses represent standard error by participants, corrected for between-participant variance (Bakeman and McArthur, 1996).

#### Comprehension

As in Experiment 1, one participant was removed from further analysis due to low accuracy (less than 70% accurate). Accuracy on the comprehension questions averaged 84% across participants, indicating that the participant attended to the stimuli. Across participants, accuracy ranged from 71 to 100%.

#### Reading times

**Figure 2:**
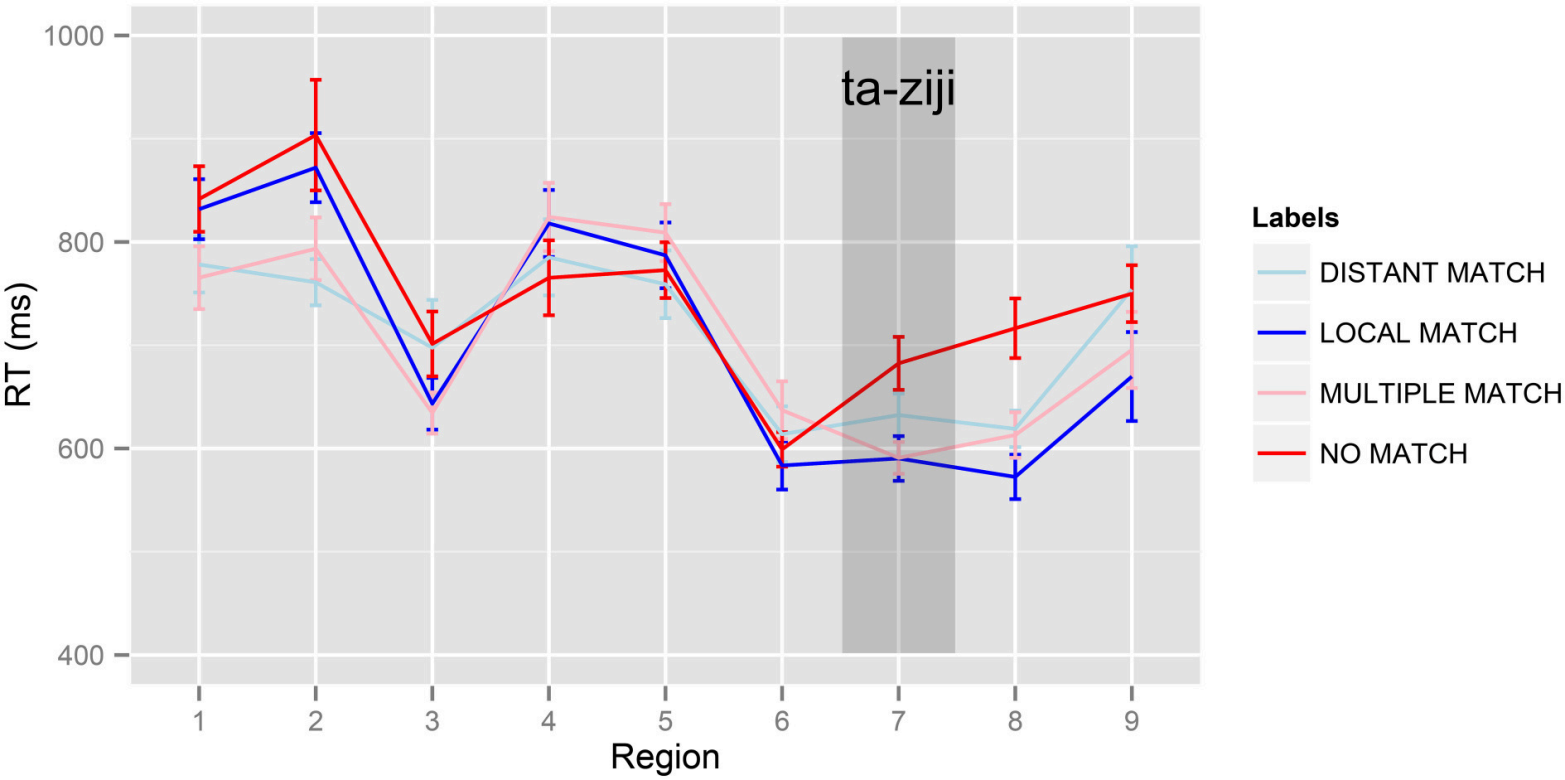
**Mean reading times per region in Experiment 2**. Error bars represent standard error by participants, corrected for between-participant variance (Bakeman and McArthur, 1996). 1:*Zhang taitai*2:*jingchang guanggu de* 3:*na-ge* 4:*shizhuangdian* 5:*shang-ge-xingqi* 6:*ba* 7:*ta-ziji* 8:*bu xiaoxin* 9:*nongshang-le.*

Raw mean reading times are presented in Table 4 and Figure 2. Visual inspection of the means suggests a weaker locality effect in Experiment 2 than in Experiment 1: the difference between the *LOCAL MATCH* and *DISTANT MATCH* conditions was 57 ms at the critical region, and 53 ms at the spillover region (compared to 215 ms in Experiment 1). The reading times suggest a numerically an effect of the *No Antecedent contrast* (109 ms at the critical region, 148 ms in the spillover region), and a small effect for the *Multiple Match contrast* (10 ms at the critical region, 50 ms in the spillover region).

**Table 4:** **Mean reading times per region in Experiment 2**.

	**1**	**2**	**3**	**4**	**5**	**6**	**7**	**8**	**9**
*LOCAL MATCH*	832 (29)	872 (33)	643 (25)	818 (32)	787 (32)	584 (23)	590 (22)	572 (22)	670 (43)
*DISTANT MATCH*	778 (27)	761 (22)	698 (46)	785 (37)	759 (33)	614 (27)	632 (21)	619 (18)	754 (42)
*MULTIPLE MATCH*	765 (30)	793 (30)	634 (20)	824 (33)	809 (28)	637 (28)	591 (15)	613 (22)	695 (37)
*NO MATCH*	842 (32)	904 (54)	701 (31)	765 (36)	773 (27)	599 (17)	682 (26)	716 (29)	750 (28)

Parentheses represent standard error by participants, corrected for between-participant variance (Bakeman and McArthur, 1996). 1:Zhang taitai 2:jingchang guanggu de 3:na-ge 4:shizhuangdian 5:shang-ge-xingqi 6:ba 7:ta-ziji 8:bu xiaoxin 9:nongshang-le.

The results of the statistical modeling of the reaction times at the pre-critical, critical, and spillover regions are presented in Tables 5–7. Analysis revealed no significant effects of counterbalancing list, and so we report models that do not include list as a fixed effect predictor.

**Table 5:** **Experimental fixed effects estimates from linear mixed effects modeling of pre-critical region across Experiments 1 and 2**.

	**Estimate**	***t***
Experiment	0.09 (0.06)	1.45
*LOCALITY*	−0.02 (0.05)	−0.40
*NO MATCH*	0.04 (0.04)	0.90
*MULTIPLE MATCH*	0 (0.05)	0.10
Experiment: *LOCALITY*	0.03 (0.06)	0.49
Experiment: *NO MATCH*	−0.01 (0.06)	−0.17
Experiment: *MULTIPLE MATCH*	0.06 (0.06)	1.00

**Table 6:** **Experimental fixed effects estimates from linear mixed effects modeling of critical region across Experiments 1 and 2**.

	**Estimate**	***t***
Experiment	0.24 (0.06)	4.30
*LOCALITY*	0.06 (0.05)	1.26
*NO MATCH*	0.05 (0.04)	1.02
*MULTIPLE MATCH*	0 (0.04)	0.12
Experiment: *LOCALITY*	−0.02 (0.05)	−0.38
Experiment: *NO MATCH*	0.05 (0.05)	0.91
Experiment: *MULTIPLE MATCH*	0.02 (0.05)	0.38

**Table 7:** **Experimental fixed effects estimates from linear mixed effects modeling of spillover region across Experiments 1 and 2**.

	**Estimate**	***t***
Experiment	0.13 (0.06)	2.03
*LOCALITY*	0.29 (0.05)	6.31
*NO MATCH*	0.14 (0.04)	3.05
*MULTIPLE MATCH*	0.05 (0.05)	1.08
Experiment: *LOCALITY*	−0.22 (0.06)	−3.94
Experiment: *NO MATCH*	0.03 (0.06)	0.52
Experiment: *MULTIPLE MATCH*	0.01 (0.05)	0.14

At the pre-critical region, *ba*, we did not observe any statistically significant effects. This pattern suggests any early differences in the materials between conditions—such as the animacy of the subject, or the different relative clauses used in different conditions—had returned to a neutral baseline prior to the critical region. We examined this observation further by performing an additional analysis of the region that immediately preceded the pre-critical region (e.g., *shangge xingqi*, “last week”). As in the pre-critical region, we failed to observe any statistically significant effects, providing further evidence that pre-critical differences in the materials across conditions did not have durable or long-lasting effects on reading times preceding the critical region.

In the critical region, we observed only a fixed effect of *Experiment*. Reading times in the anaphor region were significantly longer in Experiment 2 compared to Experiment 1, presumably reflecting the fact that *ta-ziji* is longer than *ziji*.

**Figure 3:**
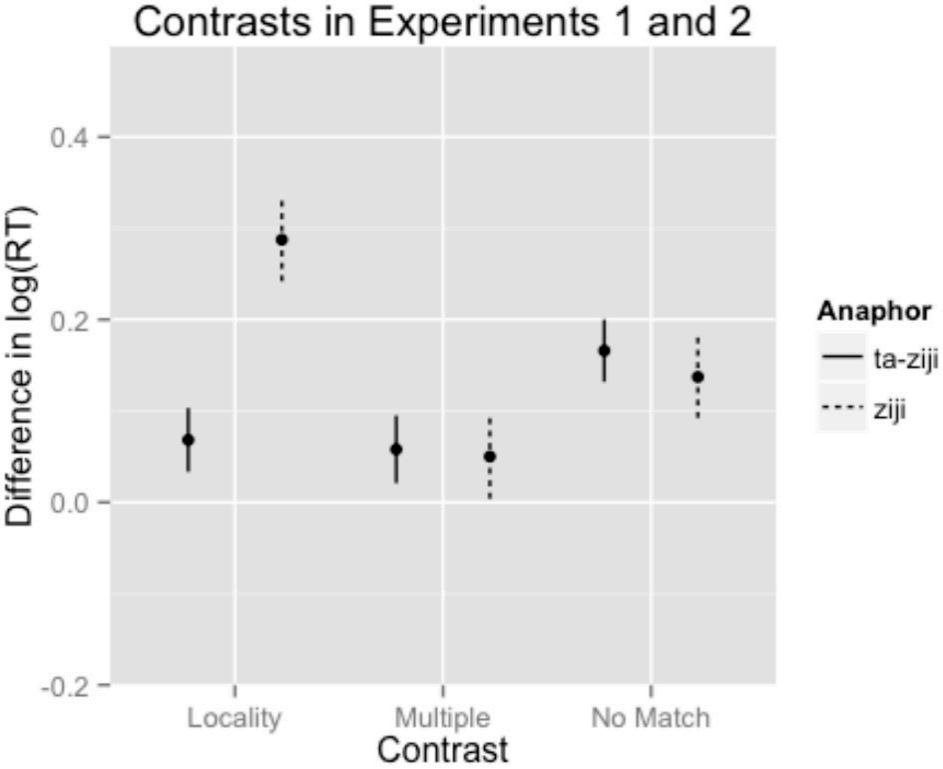
**Fixed effects estimates for Experimental contrasts in the spill-over region in both Experiment 1 (*ziji*) and Experiment 2 (*ta-ziji*)**. Error bars indicate the standard error associated with the fixed effect estimate.

In the spillover region, we observed a statistically significant effect of the *Locality contrast*, and a statistically significant effect of the *No Antecedent contrast*. We did not observe any significant effects of antecedent frequency or length. Critically, we observed an interaction of *Experiment* with the *Locality contrast*. The direction of this coefficient indicates that the locality contrast was significantly smaller in Experiment 2 than it was in Experiment 1. To further investigate the interaction of *Experiment* with the *Locality contrast*, we fit a second model in which the critical *Locality contrast* was nested within individual levels of *Experiment*. This model revealed that there was a significant *Locality contrast* for Experiment 1 (0.29 (0.05), *t* = 6.31). In Experiment 2, the estimated size of this effect was much smaller than in Experiment 1, and it was only marginal for Experiment 2 (0.07 (0.04), *t* = 1.95). The magnitude of the *No Antecedent contrast* was comparable between Experiments 1 and 2, and it reached statistical significance in both Experiments [Experiment 1: 0.14 (0.04), *t* = 3.05; Experiment 2: 0.17 (0.03), *t* = 4.85]. The *Multiple Match contrast* did not reach significance in either Experiment, although the magnitude of the observed effect and its sign were comparable across experiments [Experiment 1: 0.05 (0.05), *t* = 1.08; Experiment 2: 0.06 (0.04), *t* = 1.56]. The estimates of the fixed effects contrasts by *Experiment* yielded by this model are presented in Figure 3.

### Discussion

The offline judgments for sentences containing *ta-ziji* revealed that the *DISTANT MATCH* and *LOCAL MATCH* conditions were considered equally acceptable, and that both were considered more acceptable than the *NO MATCH* condition. This pattern confirms that the distant subject position is accessible as an antecedent for *ta-ziji* in our materials. Furthermore, this pattern gives no indication of a locality bias in the offline judgments for *ta-ziji*. This contrasts sharply with the clear offline locality bias observed for *ziji* in Experiment 1.

Note furthermore that our *DISTANT MATCH* and *LOCAL MATCH* conditions differed in whether the main clause subject was animate. As the *DISTANT MATCH* condition was rated equally highly as the *LOCAL MATCH* condition in this experiment, one may infer that the inanimate main clause subjects in the *DISTANT MATCH* condition did not impact the naturalness of the sentences. This further suggests that the difference we observed between *DISTANT MATCH* and *LOCAL MATCH* in the judgments and reading times in Experiment 1 reflect aspects of the processing of *ziji*, rather than unacceptability that results from the presence of inanimate main clause subjects in the *DISTANT MATCH* condition.

Turning to the reading times, statistical modeling of the results yields several important insights. First, although both the *Locality contrast* was significant at the spillover region for both *ziji* and *ta-ziji*, there was a significant interaction of *Locality* and *Experiment*: the magnitude of the locality effect was much smaller for *ta-ziji* than for *ziji*. Although the locality effect was several times smaller for *ta-ziji* than for *ziji, post-hoc* analysis revealed that the *Locality contrast* was significant for both anaphors.

However, apart from this crucial difference, the processing of both anaphors was qualitatively similar. Our analysis revealed a significant *No Antecedent contrast* that did not differ in magnitude across studies. This indicates that comprehenders did indeed try to assign a referent to the anaphor upon encountering it, and moreover, it suggests that comprehenders experienced a similar amount of processing difficulty when there was no sentence-internal antecedent for both *ziji* and *ta-ziji*. Likewise, the magnitude of the *Multiple Match contrast* was similar across the two experiments, although it failed to reach statistical significance either in the omnibus analysis, or in the *post-hoc* analyses by experiment.

## General discussion

In two self-paced reading experiments, we investigated the processing of two reflexive anaphors in Mandarin: the bare monomorphemic reflexive *ziji*, and the morphologically complex reflexive *ta-ziji*. In both offline acceptability rating and online reading time results, we observed that *ziji* was associated with a robust locality bias. Non-local interpretation of *ziji* was associated with lower naturalness ratings and longer reading times. In contrast, we observed a significantly smaller locality effect for *ta-ziji* in reading times, and no locality effect in offline acceptability judgments. Interestingly, this was the only difference we observed between *ziji* and *ta-ziji*. For both anaphors, we observed reliable reading time slowdowns when there was no licit antecedent in the sentence, and the size of this *no match* penalty did not reliably differ between anaphors in reading time measures. Likewise, for both anaphors we observed a trend toward a multiple match penalty. This effect did not reach statistical significance, although the consistency of the effect in sign and magnitude across experiments raises the possibility that the failure to observe this effect reflects a lack of statistical power. We take up each of these effects in turn.

### Feature richness and antecedent search

Our findings suggest that the locality bias that is associated with *ziji* does not generalize to other Mandarin reflexives that can take antecedents outside of their immediate tensed clause. Specifically, the morphologically complex anaphor *ta-ziji* shows a much diminished locality bias in online processing measures. One plausible hypothesis about this difference is that the overt morphological feature content on *ta-ziji* leads to faster or more reliable access to structurally distant antecedents. In contrast, *ziji* has fewer overt morphological cues to its antecedent, and so comprehenders may need to rely more heavily on positional cues to isolate its antecedent in memory, leading to relatively more pronounced locality bias.

This hypothesis is plausible given existing theories of how comprehenders access information in working memory during sentence comprehension. For a wide range of linguistic dependencies, there is evidence that the processor makes use of a content-addressable retrieval mechanism to form syntactic and referential dependencies between temporally distant phrases (McElree, 2000, 2006, 2014; McElree et al., 2003; Lewis and Vasishth, 2005; Lewis et al., 2006; McElree, 2006; Van Dyke and McElree, 2006, 2011; Foraker and McElree, 2011). A content-addressable retrieval mechanism accesses a representation in memory using the inherent features of the representation as cues to guide memory retrieval process. For example, a pronoun like *him* may be said to retrieve its antecedent by using gender features as cues to locate an antecedent in memory (e.g., Foraker and McElree, 2007). These cues are said to provide *direct access* to the desired representation, obviating any need to search through irrelevant representations at retrieval. This mechanism has the benefit of granting extremely rapid access to information in memory, making this an attractive mechanism for memory access in the human sentence processor (Lewis et al., 2006). In general, models that posit a content-addressable retrieval mechanism predict that an increase in structural or linear distance between the retrieval site (e.g., the anaphor) and the target of retrieval (e.g., its antecedent) may lead to reduced retrieval accuracy, but that structural or linear distance *per se* should not result in longer retrieval times. On some theoretical proposals, the speed of retrieval may be modulated by variables such as retrieval interference and temporal decay (Lewis and Vasishth, 2005; Lewis et al., 2006); on others, these variables primarily impact the probability of successfully recovering a target representation (McElree, 2006). Although cue-based models have the advantage of offering rapid access to representations in memory when they are required for sentence comprehension, they encounter difficulty if multiple representations in memory match the retrieval cues used at retrieval. If this occurs, it may be more difficult to isolate the *target* representation in memory, a phenomenon known as *retrieval interference*. Retrieval interference has been shown to be a primary cause of difficulty in sentence comprehension (Lewis, 1996; Van Dyke and Lewis, 2003; Lewis and Vasishth, 2005; Lewis et al., 2006; Van Dyke and McElree, 2006; Van Dyke, 2007; see also Gordon et al., 2001, 2002, 2004; for a recent review, see Van Dyke and Johns, 2012).

Several distinct hypotheses that draw upon this basic framework have been proposed to explain the locality bias associated with *ziji*. Chen et al. (2012) and Jäger et al. (2015) offered an account that draws on the ACT-R model of Lewis and Vasishth (2005). This model explains the locality bias as the result of decay and interference reducing the activation level of the distant subject, which in turn leads to longer antecedent retrieval times when the antecedent is distant from the anaphor. An alternative explanation is offered by Dillon et al. (2014), who proposed that the locality effect arises because comprehenders tend to initially retrieve the local subject as an antecedent for *ziji*. On this view, comprehenders must reject the local subject as a plausible antecedent and execute additional memory retrievals to access a distant antecedent for *ziji*. On this view, more retrieval operations are necessary to access distant antecedents, and so it is predicted that processing times should increase whenever *ziji* needs to take an antecedent other than the most local one.

One finding that distinguishes these two accounts is the MR-SAT study reported by Dillon et al. (2014). Dillon and colleagues observed that *ziji* with distant antecedents led to significantly slower processing rates in the speed-accuracy tradeoff function than *ziji* with local antecedents. Standard memory variables such as temporal decay and interference alone have not been shown to modulate processing speed in the speed-accuracy tradeoff functions associated with linguistic processing (McElree, 2000; McElree et al., 2003; Foraker and McElree, 2007, 2011; Martin and McElree, 2008, 2009, 2011; Van Dyke and McElree, 2011). However, processing speed as measured in the speed-accuracy tradeoff function has been shown to be slowed down by increasing the number of required retrieval operations, and in situations where syntactic reanalysis is required (McElree et al., 2003; Bornkessel et al., 2004; Foraker and McElree, 2007). Thus, the MR-SAT data lend support to the view that the locality effect arises because comprehenders are tempted to initially retrieve and evaluate the local subject as an antecedent when processing *ziji*.

This account also offers some insight into how the overt feature content of *ta-ziji* may allow comprehenders to overcome locality bias in comprehension. Many different implementations of cue-based retrieval mechanisms predict that the more highly specified a retrieval probe is in terms of the cues used, the less likely it is that partially matching (*distractor*) items in memory will cause retrieval interference, and compete with other representations at retrieval (Lewis and Vasishth, 2005; Van Dyke and McElree, 2006; Van Dyke, 2007). These models hold that retrieval probes that contain a greater number of retrieval cues will see a corresponding increase in the probability of recovering a target item in memory, because more numerous and specific retrieval cues will in general decrease the probability of retrieving a distractor that only matches a subset of the retrieval cues.

To illustrate how these models would account for the difference in the magnitude of the locality effect for *ziji* and *ta-ziji*, consider again the critical configuration in (9):

(9)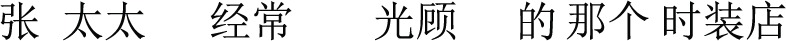  Zhang taitai_i_ jingchang guanggu de na-ge shizhuangdian_j_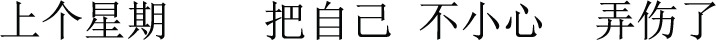  shang-ge-xingqi ba ziji_*i*/*j_ bu xiaoxin nongshang-le.
  Mrs. Zhang often visit de that-cl boutique last-week ba self not careful harm-perf.
  “The boutique that Mrs. Zhang often visits carelessly harmed her last week.”

Upon reaching the anaphor, comprehenders will, by hypothesis, recruit a mixture of syntactic cues (e.g., cues to subjecthood, such as syntactic case) and semantic or morphological cues (e.g., animacy in the case of *ziji*; animacy, humanness, and perhaps gender in the case of *ta-ziji*). Although these cues form a perfect match to the target antecedent, the inappropriate local subject *shizhuangdian* (boutique) matches only the syntactic cues. Thus, it is possible for the local antecedent to be mis-retrieved some proportion of the time, because it partially matches the syntactic cue content of the retrieval probe. Although we might reasonably expect the semantically appropriate long-distance antecedent to outcompete the local subject at retrieval in many cases, the competition contributed by the local subject may be exacerbated by its recency or its structural proximity to the anaphor (Dillon et al., 2014). If the retrieval probe contains relatively few semantic cues, the likelihood of mis-retrieving the local subject may be relatively high. On the account offered by Dillon et al. (2014), this is precisely what happens when comprehenders process *ziji*: although *ziji* contains animacy cues, these are not enough to overcome interference from the local subject, and so the local subject is retrieved some proportion of the time. When this occurs, comprehenders must attempt additional retrievals in order to arrive at an acceptable interpretation of the anaphor.

In the case of *ta-ziji*, the addition of humanness and gender features into the retrieval probe ensures that the local subject *na-ge shizhuangdian* “that boutique” matches fewer retrieval cues in the probe. This decreases the probability of retrieving the partially matching local subject, resulting in a greater proportion of trials when comprehenders are able to access the desired antecedent without sampling multiple antecedent representations from memory. Thus, if the locality bias reflects a tendency to mis-retrieve and consider the local subject, rather than decay of the distant antecedent *per se*, then the locality bias is predicted to be smaller for *ta-ziji* than for *ziji*. Put simply, *ta-ziji*'s additional feature content decreases the attractiveness of the local subject as a distractor and makes it more likely that comprehenders will successfully retrieve the long-distance antecedent on their first attempt.

Although, we have offered an explanation of our results in terms of the likelihood that the local subject will be (mis-)retrieved when processing the anaphor, it remains to be seen whether accounts that explain the locality bias effect as decreased activation of the distant antecedent can account for the difference between *ziji* and *ta-ziji*. The contrast between these anaphors rules out the simple hypothesis that the locality bias associated with *ziji* is due to recency or temporal decay alone. This is because this hypothesis would predict an equal locality effect for both anaphors. However, it may be possible to capture the present finding in more sophisticated models where the activation of an item in memory is partially a function of the retrieval cues used to access memory, such as the ACT-R model of Lewis and Vasishth (2005). It is difficult to evaluate the predictions of these models without the aid of an implemented computational model. The predictions of this account vary substantially with specific modeling assumptions that one makes. For example, if one assumes that the distant antecedent in examples like (9) is a perfect match to the retrieval probe of *ziji* and *ta-ziji* alike, then the activation of the distant antecedent should not be modulated by the number of retrieval cues in the retrieval probe^1^. This is because the total activation boost that a retrieval probe gives to an item in memory is constant in ACT-R. Adding more retrieval cues to the probe therefore does not increase the amount of activation afforded to a perfectly matching item in memory; it instead diminishes the amount of activation boost that is contributed by any one cue on its own. Under these conditions, availability of the distant antecedent is not predicted to differ between *ziji* and *ta-ziji*, all else being equal. However, if one relaxes these assumptions, it may be possible to capture this result. Thus, although we cannot claim that the present results are incompatible with the explanation of the locality bias effect offered by Chen et al. (2012) and Jäger et al. (2015), more research and modeling work is necessary to determine the specific circumstances under which these models can capture the contrast between *ziji* and *ta-ziji*.

### *Ziji* vs. *ta-ziji*

The explanation we offer for our findings posits that overt morphological features provide retrieval cues for recovering an antecedent for an anaphoric expression. However, the precise relationship between overt morphological form and the cues used to retrieve an antecedent remains unclear. Indeed, the problem of specifying the nature of the retrieval cues that support language processing is a key theoretical issue for cue-based approaches, and remains an area where much further work is needed (Van Dyke and McElree, 2011; Dillon et al., 2013). Previous research suggests that the link between overt morphological feature content and retrieval cues may be rather indirect, such that there is not a one-to-one mapping between overt morphological features and retrieval cues. For example, Dillon et al. (2013) presented a series of studies that investigated the processing of English reflexive *himself*. On the basis of a comparison between computational retrieval models and online reading time data, they suggest that *himself* does not use gender and number features as cues to retrieve its antecedent, instead relying on a mixture of structural and locality cues (Dillon et al., 2013; Dillon, 2014; but see Jäger et al., 2015, for a critical view of this conclusion and an alternative analysis of these findings). Put differently, Dillon et al. (2013) proposed that the morphologically complex English reflexive *himself* deploys a cue set that is fundamentally similar to the cue set proposed for Mandarin *ziji*, despite the fact that *himself* is morphologically more similar to Mandarin *ta-ziji* than it is to *ziji*. This contrast suggests that the simple hypothesis that overt morphological features are recruited as retrieval cues may not be correct, but we presently lack a theory of how morphological and semantic features of anaphoric expressions are used during antecedent retrieval.

Resolving this tension is beyond the scope of this paper, but there are several possibilities that suggest themselves. It may be that the direct link between morphology and retrieval cues that we offer as an explanation for the present findings is misguided, and some other difference between *ziji* and *ta-ziji* is responsible for the difference in their processing behavior. One plausible alternative explanation of our result could leverage the observation that *ta-ziji* may be readily interpreted as a contrastive reflexive, analogous to *he himself* in English (Pan, 1998; what Baker, 1995 calls an *intensive pronoun*). Baker (1995) suggests that the interpretive constraints on these intensive pronouns are best understood in terms of discourse prominence and contrastiveness of potential antecedents, rather than their syntactic positions. It is possible that the diminished locality effect for *ta-ziji* reflects a preference to construe *ta-ziji* as an intensive pronoun in our experiment. This could have caused readers to weight discourse cues more heavily than syntactic cues when retrieving an antecedent for *ta-ziji*, leading to a smaller locality effect. While we find this an interesting possibility, we cannot confidently endorse it on the basis of the present data because it is at present unclear whether readers understood *ta-ziji* as an anaphor or an intensive pronoun in our experiment. Another possibility is that the difference in locality bias reflects a difference in the frequency with which each anaphor takes antecedents beyond its local clause. Although we cannot rule out this possibility, we find it unlikely: the number of syntactic environments where *ta-ziji* can find an antecedent outside of its tensed clause is much smaller than the number of environments where *ziji* can, making it unlikely that *ziji* more often takes a local antecedent than *ta-ziji* in a Mandarin speaker's language experience. Nonetheless, corpus work would be necessary to secure this conclusion, and at present we must regard it as a possible, but unlikely, explanation of the present finding.

### Multiple match effects

A further prediction of cue-based retrieval models is that the presence of multiple antecedents that match the retrieval cues of the anaphor should create retrieval interference, which should in turn create processing difficulty. For example, consider our *MULTIPLE MATCH* condition:

(10)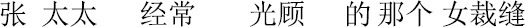  Zhang taitai_*i*_ jingchang guanggu de na-ge nücaifeng_*j*_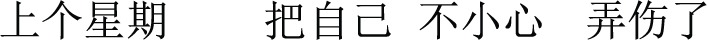  shang-ge-xingqi ba ziji_*i/j_ buxiaoxin nongshang-le.
  Mrs. Zhang often visit de that-cl seamstress last-week ba self not careful harm-perf.
  “The seamstress that Mrs. Zhang often visits carelessly harmed her last week.”

This sentence is not ambiguous, as the animate head noun *nücaifeng* (“seamstress”) blocks access to the embedded subject *Zhang taitai* (“Mrs. Zhang”; see Tang, 1989). In other words, *Zhang taitai* is a grammatically inaccessible distractor in this example, even though this syntactic position would have been grammatically accessible if the relative clause's head noun were inanimate. Nonetheless, because the distant subject *Zhang taitai* matches the retrieval cues of the anaphor, it is predicted to create retrieval interference. Because there are multiple antecedents that match the animacy cues associated with *ziji*, it should be more difficult for comprehenders to isolate the correct antecedent *nücaifeng* in memory. In terms of our experimental manipulations, cue-based parsing models broadly predict that the *MULTIPLE MATCH* condition should be more difficult than the *LOCAL MATCH* condition at the reflexive, because the *MULTIPLE MATCH* condition contains more representations that match the retrieval cues, contributing to retrieval interference in the *MULTIPLE MATCH* condition that should inhibit access to the target antecedent. Moreover, it is predicted that the size of the multiple match effect should be greater for *ta-ziji* than for *ziji*, because the distractor matches a greater proportion of the retrieval cues for the complex anaphor.

Our experiments failed to provide clear evidence to support or disconfirm these predictions. In both Experiments 1 and 2, we observed numerically longer reading times on the *MULTIPLE MATCH* condition than in the *LOCAL MATCH* condition, but this contrast did not reach statistical significance in either experiment alone, and we failed to observe any trend toward a larger multiple match effect for *ta-ziji*. The interpretation of the present findings, and their relationship with previous findings, must therefore be treated with caution. We note that the size and magnitude of the multiple match effect was consistent, and in the predicted direction, in both experiments. This pattern suggests that our failure to find an effect may reflect a lack of statistical power.

Although a multiple match penalty has been observed in previous reading time studies (Badecker and Straub, 1994, 2002; Kennison et al., 2003; Felser et al., 2009; Chen et al., 2012; Jäger et al., 2015; see also Rigalleau et al., 2004; Stewart et al., 2007), the empirical generalization has remained unclear, especially for reflexive pronouns. Although some studies have presented evidence that feature-matched, but structurally inaccessible antecedents create processing difficulty for reflexive pronouns (Badecker and Straub, 2002; Felser et al., 2009; Chen et al., 2012; Jäger et al., 2015), many more studies have failed to find reliable evidence for such an effect in reading time measures, or found it only in limited contexts (Clifton et al., 1999; Sturt, 2003; Xiang et al., 2009; Cunnings and Felser, 2013; Dillon et al., 2013; Cunnings and Sturt, 2014; Kush and Phillips, 2014; Jäger et al., 2015). It is notable that Chen and colleagues reported that reading times on *ziji* were longer in the presence of a grammatically inaccessible, but semantically appropriate antecedent (c.f., Jäger et al., 2015). On balance, however, the repeated failures to find multiple match effects suggest that grammatically illicit antecedents do not create substantial interference effects, and so our failure to find any multiple match effects in the present study may not be surprising. Dillon (2014) and Sturt (2013) offer reviews of the empirical landscape, and suggest that grammatical constraints act as strong filters on antecedent retrieval, allowing for very little (if any) retrieval interference from grammatically illicit antecedents.

A similar pattern emerges for studies that have focused on the processing of direct object pronouns in English. Chow et al. (2014) reported five experiments that sought to find multiple match effects with direct object pronouns in English, and failed to find any evidence of such an effect (including a near direct replication of Badecker and Straub, 2002). On the basis of this finding, Chow and colleagues argued that, in line with the processing of reflexives, structural constraints acted immediately to help rule out grammatically inaccessible antecedents for object pronouns as well (see also Clifton et al., 1997; Lee and Williams, 2008; Patterson et al., 2014).

On the basis of the non-significant multiple match effects in the present studies, very little can be concluded about whether the inaccessible antecedent in our *MULTIPLE MATCH* conditions created retrieval interference. However, inconsistent with claims that grammatical constraints rule out inaccessible antecedents, we did find clear trends in the predicted direction in both experiments. We thus regard it as an open empirical question whether an animate distant subject interferes with the retrieval of the correct local subject when processing *ziji* and *ta-ziji*.

## Conclusion

In our Experiment 1, we observed that the morphologically simple long-distance reflexive *ziji* showed a robust locality bias in reading time measures. Experiment 2 revealed that the morphologically complex, local reflexive *ta-ziji* showed a much reduced locality bias in processing. We proposed that this contrast was due to the number of morphological and semantic features each anaphor uses during the process of retrieving an antecedent. Morphologically simple anaphors like *ziji*, which have relatively fewer retrieval cues, are more likely to access non-target antecedents at retrieval. This requires comprehenders to sample multiple antecedents in order to achieve an interpretation for the anaphor, leading to locality effects. In contrast, the relatively more specified *ta-ziji* has more cues for antecedent retrieval, which makes it less susceptible to interference from non-target representations. For this reason, complex anaphors like *ta-ziji* show diminished locality effects in comprehension.

### Conflict of interest statement

The authors declare that the research was conducted in the absence of any commercial or financial relationships that could be construed as a potential conflict of interest.

## Footnotes

^1^We are grateful to Lena Jäger for this observation.
